# Spike protein–induced VSIR–ISX signaling disrupts metabolic homeostasis and promotes COVID-19–related immune dysfunction

**DOI:** 10.1007/s10565-025-10119-2

**Published:** 2025-12-19

**Authors:** Li-Ting Wang, Shen-Nien Wang, Shyh-Shin Chiou, Chee-Yin Chai, Shih-Hsien Hsu

**Affiliations:** 1 Department of Life Science, National Taiwan Normal University, Taipei, Taiwan; 2 Division of General and Digestive Surgery, Department of Surgery, Kaohsiung Medical University Hospital, Kaohsiung, Taiwan; 3 Graduate Institute of Medicine, College of Medicine, Kaohsiung Medical University, Kaohsiung, Taiwan; 4 Department of Surgery, College of Medicine, Kaohsiung Medical University Hospital, Kaohsiung, Taiwan; 5 Division of Pediatric Hematology and Oncology, Department of Pediatrics, Kaohsiung Medical University Hospital, Kaohsiung Medical University, Kaohsiung, 807 Taiwan; 6 Graduate Institute of Clinical Medicine, Kaohsiung Medical University, Kaohsiung, 807 Taiwan; 7 Department of Pathology, College of Medicine, Kaohsiung Medical University, Kaohsiung, Taiwan; 8 Department of Medical Research, Kaohsiung Medical University Hospital, Kaohsiung Medical University, Kaohsiung, Taiwan; 9 Research Center for Precision Environmental Medicine, Kaohsiung Medical University, Kaohsiung, Taiwan; 10 Division of General and Digestive Surgery, Department of Surgery, Pingtung Hospital, Ministry of Health and Welfare, Pingtung, Taiwan

**Keywords:** COVID-19, ACE2, ISX, NF-kappaB, AA metabolic pathway

## Abstract

COVID-19 has caused millions of deaths worldwide since 2019. Vaccination has reduced both transmission and disease severity. However, emerging viral variants have weakened vaccine effectiveness, highlighting the need for new antiviral therapies. This study examines how the SARS-CoV-2-Spike protein (SARS-2-S) induces the VSIR-ISX signaling pathway, leading to metabolic disturbances that may worsen disease progression. Using RNA sequencing, we found that SARS-2-S expression in pulmonary cells activates genes involved in tryptophan and arachidonic acid (AA) metabolism, altering bioactive mediators like kynurenine and prostanoids, which are crucial for inflammation and immune responses. Mechanistically, the ACE2-MYD88 pathway, activated by SARS-2-S, enhances the VSIR-ISX axis through NF-κB signaling, driving these metabolic disruptions. Chromatin immunoprecipitation and genome sequencing revealed that ISX, activated via VSIR-MAPK signaling, upregulates enzymes involved in AA metabolism by binding directly to their gene promoters. Notably, disrupting the VSIR-ISX axis using shRNA interference or NF-κB inhibitors effectively mitigated these metabolic disturbances. Our findings suggest that the VSIR-ISX pathway could be a promising therapeutic target for treating COVID-19 by addressing virus-induced metabolic disruptions.

**Graphical Abstract:**

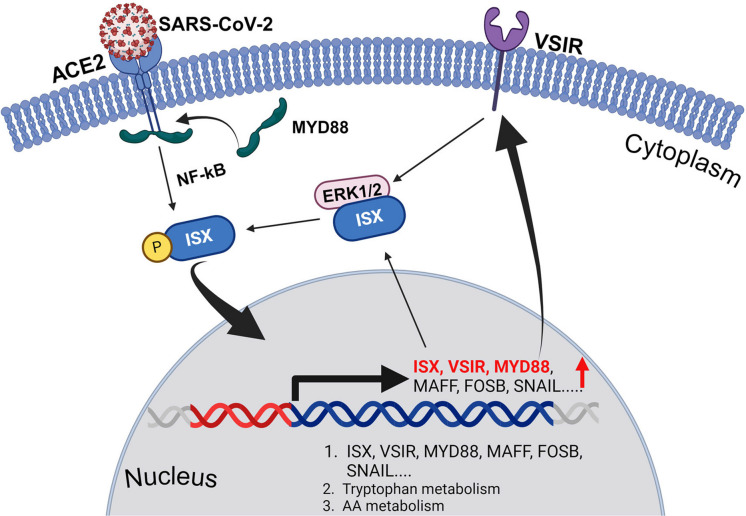

**Supplementary Information:**

The online version contains supplementary material available at 10.1007/s10565-025-10119-2.

## Introduction

Over the past three years since the onset of the COVID-19 pandemic, COVID-19 infections have profoundly changed lives and emerged as a leading cause of death worldwide (Zhu et al. 2020). The most common symptoms of COVID-19 are fever, exhaustion, pain, runny nose, and dry cough. Some patients exhibit nasal congestion, a sore throat, or diarrhea when infected with various virus mutations. Apart from vaccination, few treatments are available (Ding et al. 2020; Wang et al. 2020a).

COVID-19 is caused by infection with a novel coronavirus, SARS-CoV-2, and was first identified in December 2019 in Wuhan, China (Zhu et al 2020). SARS-CoV-2 infects the respiratory tract and causes digestive, neurological, and cardiovascular symptoms (Ding et al. 2020). Aside from vaccination, effective treatments remain limited to fight COVID-19 (Ratre et al. 2021). The Spike protein (SARS-2-S), a large and essential glycoprotein encoded by the SARS-CoV-2 genome, serves as a major viral entry factor and immune target (Lan et al. 2020). Through recognizing and interacting with surface angiotensin-converting enzyme 2 (ACE2) in the host cells, SARS-2-S initiates virus invasion, replication, and amplification (Lan et al. 2020; Ke et al. 2020). Multiple vaccines that target SARS-2-S have been developed over the last year, and the development of additional vaccines is ongoing. However, the emergence of a new SARS-2-S with immune-evading characteristics and increased infectiousness has made vaccines less effective against these variants. Consequently, we require effective and safe drugs to treat the serious symptoms of this disease.

The V-set immunoregulatory receptor (VSIR) is a recently identified novel member of the B7 immune checkpoint (IC) family of immunoregulatory genes (Wang et al. 2011) and has emerged as a promising target for combined cancer immunotherapy (Vanmeerbeek et al. 2024; Gao et al. 2024). The VSIR gene is located within an intron of the CDH23 gene on human chromosome 10 (10q22.1) and encodes a type I transmembrane protein composed of 279 amino acids (ElTanbouly et al. 2019). This protein includes an extracellular domain, a transmembrane domain, and a cytoplasmic domain. As an immune checkpoint molecule for T cells, VSIR is predominantly expressed in hematopoietic tissues and in sites with leukocyte infiltration, such as the lungs, in healthy individuals (Lines et al. 2014a, 2014b; Liu 2014). Notably, VSIR also shows high expression in human placental tissue, suggesting a potential role in allofetal tolerance (Lines et al. 2014b). Although the precise mechanisms regulating intracellular signaling by VSIR are not yet fully understood, emerging evidence indicates that BRAF/MEK signaling pathways mediate VSIR signaling, promoting tumor development and reshaping the tumor microenvironment (Rosenbaum et al. 2020). Additionally, the tumor suppressor p53 can activate the transcription of VSIR by binding to its promoter region, which facilitates the phagocytosis of malignant cells by macrophages (Lin et al. 2024; Park et al. 2020).

In this study, we aimed to identify potential therapeutic approaches for COVID-19. Our findings show that when expressed in lung cells, SARS-CoV-2 Spike protein (SARS-2-S) triggers a range of enzymes associated with metabolic disturbances, particularly in arachidonic acid (AA) metabolism. This results in altered levels of bioactive mediators such as kynurenine, prostanoids, leukotrienes (LTs), eicosatetraenoic acids (ETEs), and lipoxins (LXs). Mechanistically, we demonstrated that SARS-2-S upregulates the expression of VSIR and ISX in lung cells. Activated ISX, via VSIR-MAPK signaling, subsequently increases the expression of enzymes involved in AA metabolism by directly binding to the promoter regions of target genes. These alterations contribute to inflammation and impair immune function, which provide a novel signals and significant clinical implications for COVID-19 treatment in future.

## Results

### Transcriptomic analysis reveals dysregulated metabolic and inflammatory pathways in SARS-CoV-2–infected lung cells

**Fig. 1:**
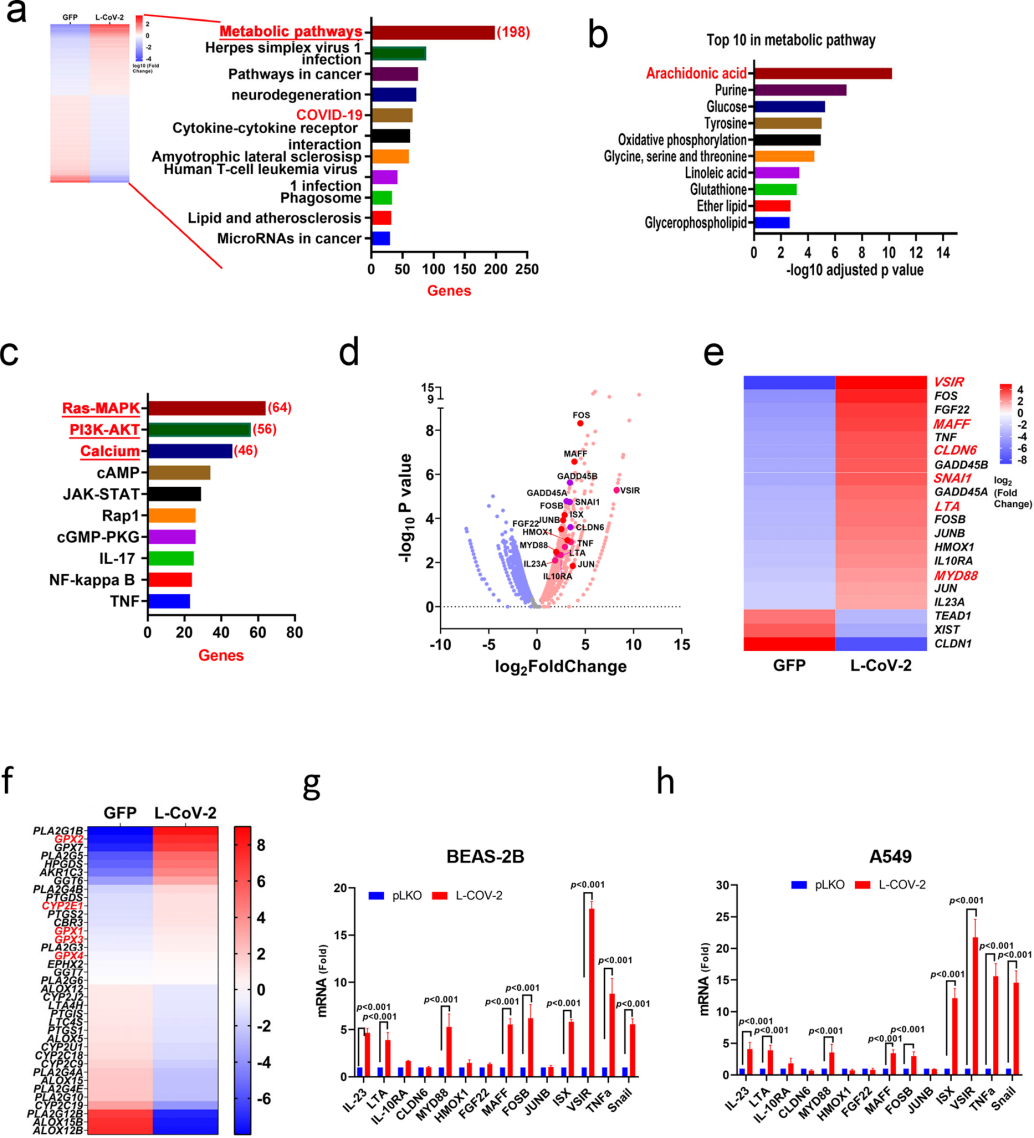
Transcriptomics analysis of lung cells infected with L-CoV-2 (**a**) Heatmap showing differentially expressed genes (DEGs) identified by RNA-seq in BEAS-2B cells with or without SARS-CoV-2 (L-CoV-2) infection. DEGs were defined as transcripts with fold change > 1.5 and false discovery rate (FDR) < 0.05 (DESeq2 analysis). (**b**) Kyoto Encyclopedia of Genes and Genomes (KEGG) enrichment analysis of significantly dysregulated metabolic pathways based on the DEG list. The top ten enriched metabolic pathways are displayed according to adjusted p-value ranking, with numbers in brackets indicating the number of enriched genes. (**c**) KEGG enrichment analysis of significantly dysregulated signaling pathways (non-metabolic) derived from the same DEG dataset. (**d**) Volcano plot displaying all differentially expressed genes (DEGs) identified by RNA-seq (fold change > 1.5, FDR < 0.05). Significantly upregulated genes are shown in red and downregulated genes in blue. Representative genes of interest—including inflammatory regulators (FOSB, JUN, IL10RA, MYD88, HMOX1) and metabolic or immune regulators (ISX, VSIR, MAFF)—are labeled for emphasis. The horizontal dashed line at y = 0 denotes the baseline corresponding to a p-value of 1 (no statistical significance). (**e**–**f**) Heatmap showing the top-ranked DEGs enriched in significantly dysregulated metabolic and signaling pathways, as determined by KEGG analysis. Expression patterns are compared between control BEAS-2B cells and BEAS-2B cells infected with L-CoV-2. (g-h) The relative mRNA expression levels of IL-10RA, IL-23, LTA4, CLDN6, MYD88, HMOX1, FGF22, MAFF, FOSB, JUNB, ISX, VSIR, TNFα, and SNAIL were measured in A549 and BEAS-2B cells infected with L-CoV-2 using semi-quantitative RT-PCR. The results are shown as the mean ± s.d., using unpaired t-tests (n = 3–5 biological replicates)

To investigate SARS-CoV-2 activities in lung cells, signals regulation was evaluated in BEAS-2B cells, with or without SARS-CoV-2 pseudotyped lentivirus (L-CoV-2) infection, by next-generation RNA-sequencing (Fig. 1a). At baseline, BEAS-2B cells showed 722 genes with significantly higher expression than controls, spanning a broad range of metabolic functions (Fig. 1a). Pathway analysis on the Kyoto Encyclopedia of Genes and Genomes (KEGG) showed that, among the differentially expressed genes in BEAS-2B cells infected with L-CoV-2, most of the pathways identified were associated with cellular metabolism (Fig. 1a). The top five differentially expressed metabolism pathways in L-CoV-2-infected BEAS-2B cells include AA, purine, glucose, tyrosine, and oxidative phosphorylation (Fig. 1b). Apart from the metabolic pathway, through KEGG analysis, top three signals, including the *Ras-MAPK, PI3K-Akt,* and calcium pathway, were also significantly activated by the L-CoV-2 infection cells (Fig. 1c). Notably, a spectrum of inflammatory and/or fibrogenic precursors were significantly activated in the L-CoV-2 infected cells, including *FOS(B), (c-)JUN, IL10RA, GADD45s, MyD88,* and *HMOX1* (Fig. 1d and e). Furthermore, levels of proto-oncogenic factors, e.g., *ISX, MAFF, FGF22,* and *SNAIL*, inflammatory cytokines, *TNF, LTA4,* and *IL-23,* and immune checkpoints molecular, *VSIR*, were also enhanced (Fig. 1e). Interestingly, key enzymes for oxidative phosphorylation and redox functions were elevated, including *GPX1-4* and *CYP2E1* (Fig. 1f). To validate the transcriptomic findings, semi-quantitative real-time RT-PCR was performed to assess the expression of representative genes across the identified pathway categories. Consistent with the RNA-seq results, inflammatory regulators (TNFα, IL-23, MYD88, HMOX1), metabolic and lipid-mediating regulators (LTA4, FGF22, MAFF), and immune checkpoint or transcriptional regulators (ISX, VSIR, SNAIL, FOSB, JUNB, CLDN6) were significantly upregulated in pulmonary cells infected with L-CoV-2 (Fig. 1g and h).

### SARS-CoV-2 activates VSIR-MAPK (ERK1/2) signaling and upregulates levels of ISX and derivatives of the AA pathway

**Fig. 2:**
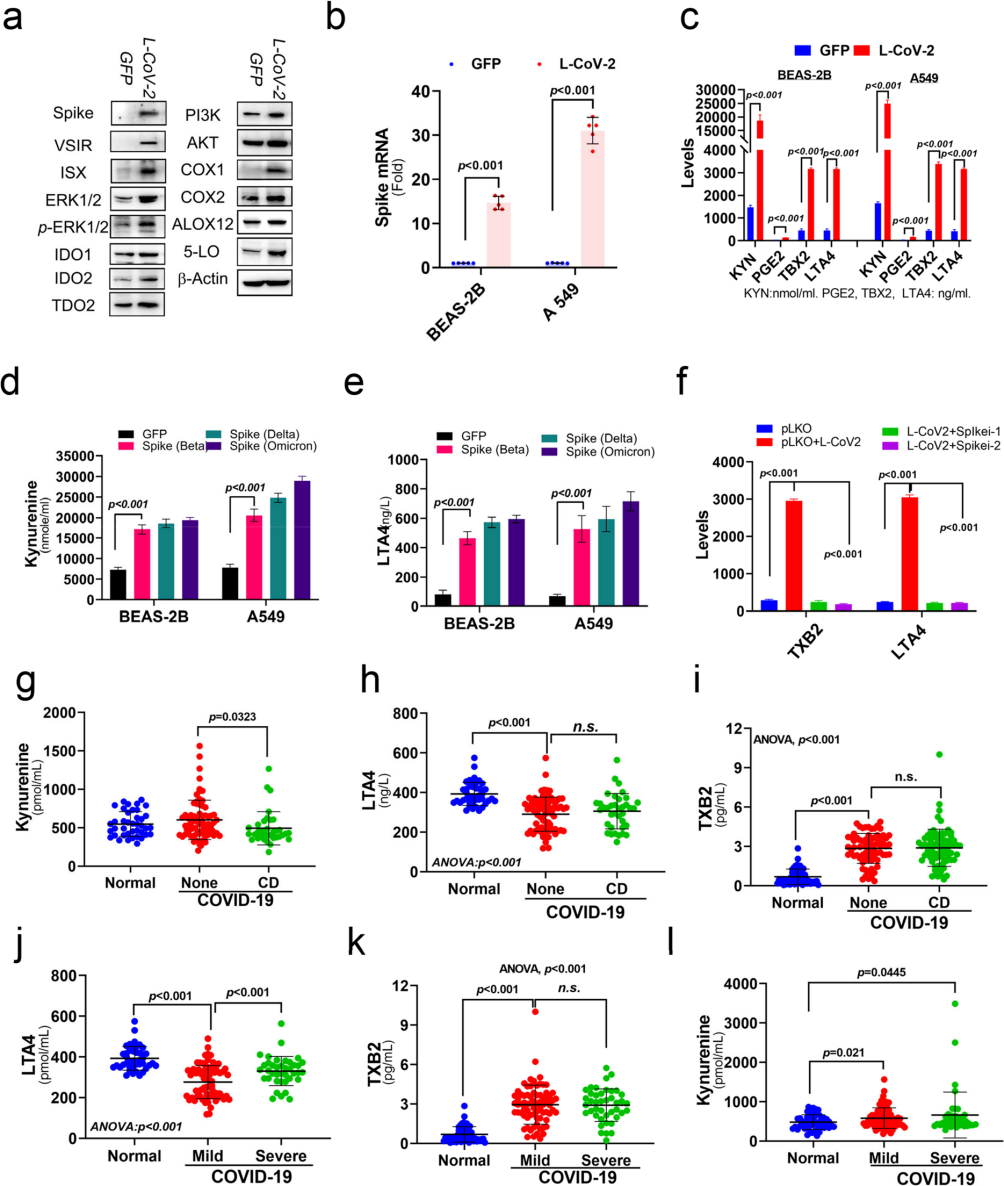
L-CoV-2 regulates metabolic pathways and inflammatory factors (**a**) The protein levels of Spike, VSIR, ISX, ERK1/2, p-ERK1/2, IDO, IDO2, TDO2, PI3K, AKT, COX1, COX2, ALOX12 and 5-lipoxygenase (5-LO) in BEAS-2B cells were analysed using Western blot. BEAS-2B cells with or without SARS-CoV-2 infection. (**b**) The relative mRNA expression levels of spike were determined in L-CoV-2–treated A549 and Base-2B cells using semiquantitative RT-PCR. (c) Kynurenine, PGE2, LTA4, and TXB2 were detected in the culture medium of BEAS-2B and A549 cells with or without L-CoV-2 infection. The results are shown as the mean ± s.d., using unpaired t-tests (n = 3–5 biological replicates). (**d**-**e**) Elevated levels of kynurenine and LTA4 in BEAS-2B and A549 cells with Spike proteins from Delta and Omicron variants. The results are shown as the mean ± s.d., using unpaired t-tests (n = 3–5 biological replicates). (**f**) LTA4 and TXB2 were detected in the culture medium of BEAS-2B and A549 cells, with or without SARS-CoV-2 infection, or in combination with spike shRNA. The results are shown as the mean ± s.d., using unpaired t-tests (n = 3–5 biological replicates). (**g**-**l**) Kynurenine, LTA4, and TXB2 were detected in the serum of patients with or without SARS-CoV-2 infection. The results are shown as the mean ± s.d., using unpaired t-tests (n = 115)

To verify the regulatory effects, cytokines and metabolites were measured in the culture supernatants, whereas protein expression in BEAS-2B cells infected with L-CoV-2 was analyzed by immunoblotting of the corresponding cell lysates. As anticipated, BEAS-2B cells infected with L-CoV-2 showed an upregulation in the levels of VSIR, ERK1/2, ISX, and AA metabolic enzymes, COX1/2, ALOX12, and 5-lipogenase (Fig. 2a and Figure S1a). Also, to validate the stimulatory effect of L-Cov-2 infection, enzyme-linked immunosorbent assay (ELISA) was used estimate the expression levels of the ISX-targeted immune modulator (Wang et al. 2017), kynurenine, and AA pathway derivatives, namely, prostaglandin E2 (PGE2), leukotriene X4 (LTA4), and Thromboxane B2 (TXB2), were assessed in these cells. As shown in Figs. 2b–c, BEAS-2B cells infected with L-CoV-2 exhibited significantly higher expression of Spike mRNA, kynurenine, and arachidonic acid (AA) pathway derivatives, including PGE2, LTA4, and TXB2, compared to cells infected with the GFP-alone control. Importantly, levels of kynurenine, PGE2, LTA4, and TXB2 in the pulmonary cells transfected with the Spike protein from different strains of the SARS-CoV-2 virus, e.g. Delta and Omicron, led to an exacerbated immune modulatory effect and AA pathway response compared to that seen in pulmonary cells infected with the beta strain of the L-CoV-2 virus (Figs. 2d–e). Notably, the L-CoV-2–induced increase in kynurenine levels was abolished in pulmonary cells transfected with Spike shRNA, which showed reduced expression of downstream mediators such as ISX and COX-2 compared with mock-transfected controls, supporting a causal role of the Spike protein (Fig. 2f).

### SARS-CoV-2 infection significantly promotes serum level of immune modulator and derivatives of AA pathway clinically

To identify the above potential pathways influenced by the SARS-CoV-2 in a clinical context, we conducted a study involving a total of 166 serum samples from patients, consisting of 51 individuals without SARS-CoV-2 infection and 115 individuals with confirmed SARS-CoV-2 infection. Our findings indicate that SARS-CoV-2 infection significantly elevates the serum metabolites levels of kynurenine, PGE2, and TXB2 but reduces the levels of LTA4 in patients without chronic diseases (CD) (Figs. 2g-i and S1b; Table 1). Interestingly, when comparing SARS-CoV-2 infected patients with chronic diseases, such as diabetes, to those without chronic diseases, we did not observe a further exacerbation in the levels of these candidate metabolites. Remarkably, SARS-CoV-2 infection led to a significant increase in the serum levels of kynurenine, PGE2, and TXB2, while decreasing the level of LTA4 in patients with both mild and severe symptoms when compared to individuals without SARS-CoV-2 infection. However, no significant difference was observed in the levels of these metabolites between SARS-CoV-2 infected patients with mild and severe symptoms (Figs. 2j-l).

**Table 1:** Baseline characteristics of COVID-19 and control cohorts

Characteristic	Non-severe (*n*=73)	Severe cohort (*n*=42)	Control group (*n*=51)	ANOVA p-value
Male/Female	38/35	21/21	34/17	-
Mean age (years)	47.6 ± 18.5	66.2 ± 12.6	51.3	-
Age range (years)	19–95	54–95	22–78	-
ICU admission (n, %)	9 (9%)	1 (6.7%)	–	-
Hospital stay (days)	16.0	30.1	–	–
Hypertension (n)	29	6	0	
Diabetes (n)	15	4	0	
CKD (n)	4	0	0	
Gout (n)	5	0	0	
No comorbidity (n)	20	32	51	
Kynurenine (pmol/mL)	602.6 ± 257.2	493.4 ± 215.9	548.3 ± 161.6	*p*=0.0596
LTA4 (ng/L)	284.9 ± 90.6	345.8 ± 119.7	393.1 ± 57.8	*p* < 0.001
TXB2 (pg/mL)	2.95 ± 1.49	2.91 ± 1.23	0.68 ± 0.59	*p* < 0.001
PGE2 (pg/mL)	403.52 ± 176.28	484.84 ± 228.73	367.26 ± 86.40	*p* = 0.0076

Data are presented as number (percentage) or mean ± standard deviation unless otherwise indicated. Statistical analysis was performed using one-way ANOVA. HTN: hypertension; DM: diabetes mellitus; CKD: chronic kidney disease; ICU: intensive care unit

### Spike–ACE2–MYD88 axis modulates kynurenine and metabolic pathways

**Fig. 3:**
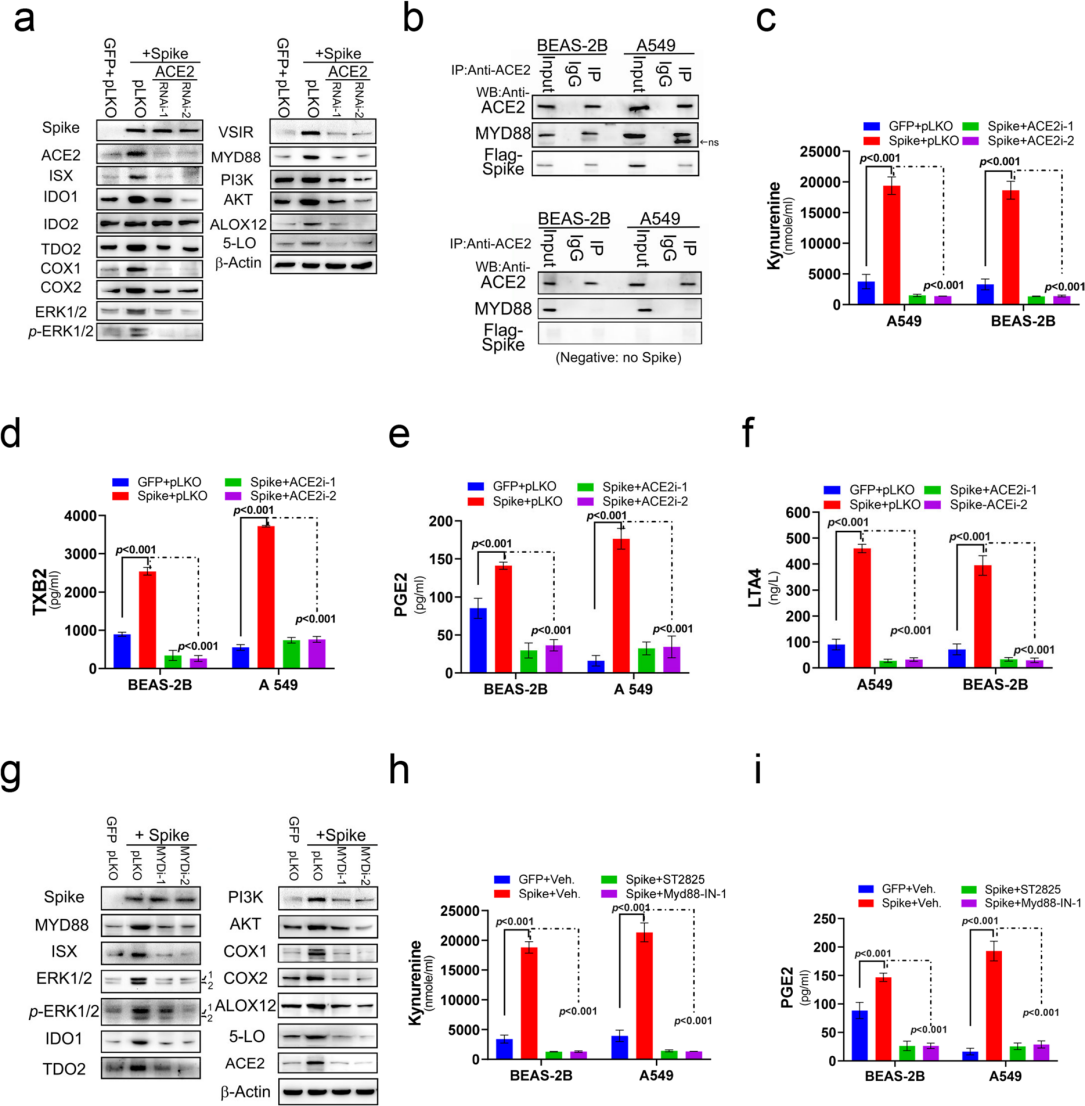
SARS-CoV-2 modulates host cells by reprogramming immune and metabolic pathways, including the arachidonic acid pathway (**a**) Protein expression levels of ISX, MyD88, immune co-inhibitors, and arachidonic acid (AA) relative signaling determined in SARS-CoV-2 regulates cells with SARS-2-S expression and transfected with or without ACE2 shRNA. (**b**) ACE2 association proteins determined by Western blot in the immunoprecipitation of BEAS-2B and A549 cells. ns, nonspecific. (**c**–**f**) Culture medium of Kynurenine, PGE2, LTA4, and TXB2 detected in BEAS-2B and A549 cells with SARS-2-S expression and transfected with or without ACE2 shRNA. (**g**) Protein expression levels of ISX, ACE2, immune co-inhibitors, and AA relative signaling determined in SARS-CoV-2 regulates cells with SARS-2-S expression and transfected with or without MYD88 shRNA. (**h**-**i**) Culture medium of Kynurenine and PGE2 detected in BEAS-2B and A549 cells with SARS-2-S expression and transfected with or without MYD88 shRNA. The results are shown as the mean ± s.d., using unpaired t-tests based on three to five biological replicates (**c**-**f**, **h** and **i**)

COVID-19 is caused by a coronavirus that anchorages on the ACE2 as the primary receptor for host cell infection (Cavezzi et al. 2022; Angeli et al. 2022). To investigate the essential impact of ACE2 on the activation of immune modulation and AA pathway induced by SARS-CoV-2-Spike protein infection, ACE2-silenced cells were established in BEAS-2B cells transfected with SARS-CoV-2-Spike to evaluate the regulatory effects of ACE2 on cellular disturbance of metabolism and immune activity. SARS-CoV-2-Spike protein activated the levels of ACE2, MYD88, immune suppressor signals (VSIR, ISX, IDO1, IDO2, and TDO2), and the key enzymes in AA metabolic pathway (COX1, COX2, ALOX12, and 5-lipoxygenase), in Ras-MAPK (ERK1/2) and in PI3K-AKT signals (PI3K and AKT) at the protein level (Fig. 3a and Figure S1c). Co-immunoprecipitation analysis demonstrated that ACE2 physically associates with MYD88 following SARS-CoV-2 Spike protein expression in BEAS-2B and A549 cells, supporting a direct signaling interaction that underlies activation of the downstream NF-κB/ISX pathway (Fig. 3b). Also, SARS-CoV-2-Spike protein promoted the metabolites levels of tryptophan and AA metabolic pathway, including kynurenine, PGE2, TXB2, and LTA4 (Fig. 3c-f). However, pulmonary cells transfected with ACE2-specific shRNA showed to abrogate the activation of ISX, ACE2, MYD88, immune suppressor factor (VSIR), the AA pathway, Ras-MAPK, and PI3K-AKT induced by SARS-CoV-2-Spike protein expression compared to mock-infected cells (Fig. 3a). As predicted, pulmonary cells transfected with SARS-CoV-2-Spike significantly promoted the levels of Kynurenine, PGE2, LTA4, and TXB2, but ACE2-silenced pulmonary cells exhibited the abolishment of Kynurenine, PGE2, LTA4, and TXB2 induced by SARS-CoV-2-Spike protein (Figs. 3c–f).

To investigate the essential impact of MYD88 on the activation of immune modulation and AA pathway induced by SARS-CoV-2-Spike protein infection, cells treated with MYD88-specific shRNA or MYD88 inhibitors (ST2825, 20 μM and Myd88-IN-1, 5 μM) were established in BEAS-2B cells transfected with SARS-CoV-2-Spike to evaluate the regulatory effects of ACE2-MYD88 signals on cellular disturbance of metabolism and immune activity. As anticipated, pulmonary cells transfected with MYD88-specific shRNAi showed to abrogate the activation of ACE2, ISX, immune suppressor factor (VSIR), the AA pathway, Ras-MAPK, and PI3K-Akt induced by SARS-CoV-2-Spike protein expression compared to mock-infected cells (Fig. 3g Figure S1d). Pulmonary cells treated with MYD88 inhibitors exhibited significantly to abolish the upregulation of Kynurenine and PGE2 induced by SARS-CoV-2-Spike protein (Figs. 3h–i).

### SARS-CoV-2-Spike regulates the ISX and consequent metabolic enzymes in AA pathway

**Fig. 4:**
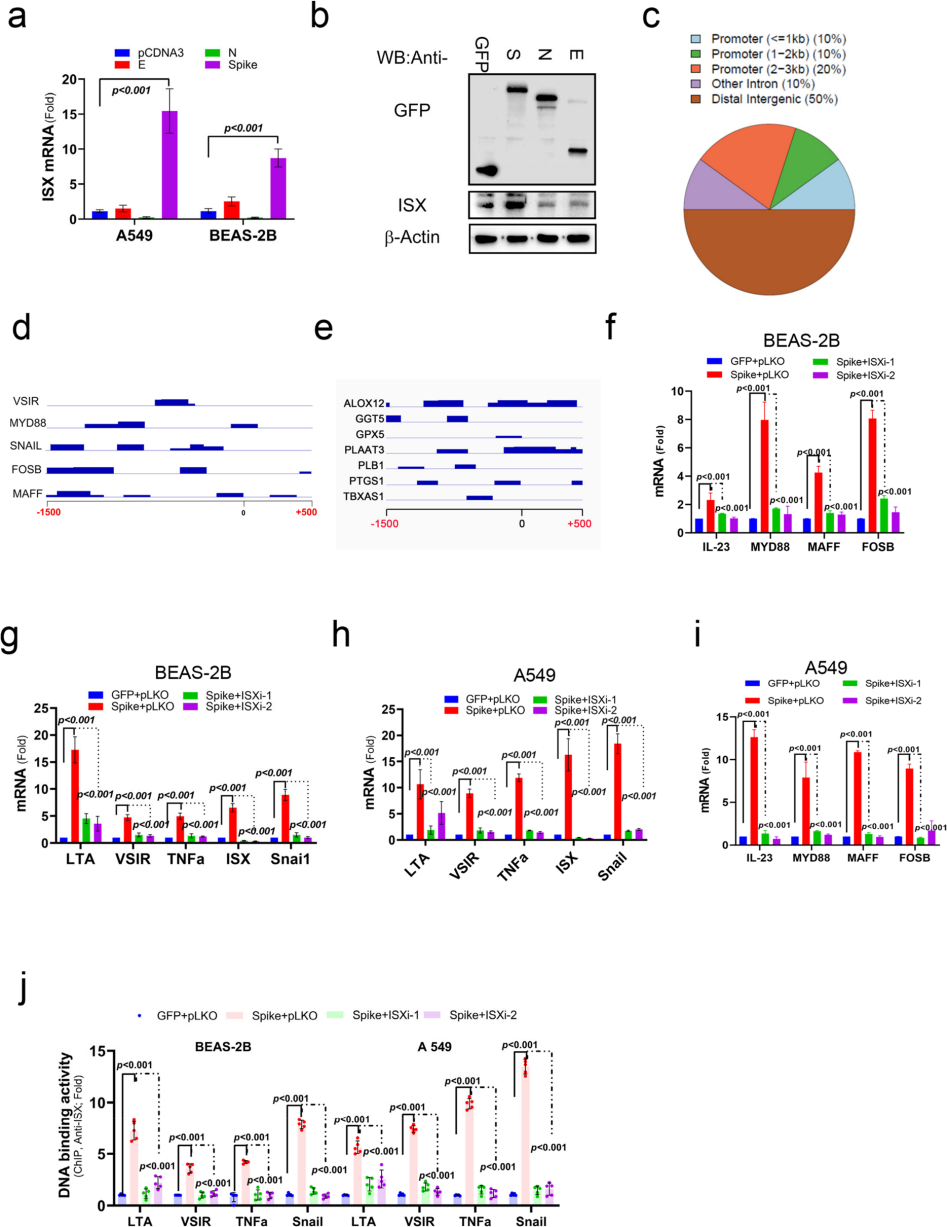
SARS-2-S promotes ISX signaling (**a**–**b**) mRNA and Protein expression levels of ISX in BEAS-2B cells transfected with SARS-2-S. S, SARS-CoV-2; E, envelope protein; N, nucleocapsid protein (**c**) Pie chart of different genomic regions. (**d**-**e**) The genomic binding fragments on the promoters of genes in VSIR, Myd88, SNAIL, FOSB, and MAFF, as well as those related to AA signaling, all have peak calling within 1 kbp of the promoter region. (**f**-**i**) The relative mRNA expression levels of IL-33, MyD88, MAFF, FOSB, LTA4, VSIR, TNFα and SNAI1 were determined in A549 and Base-2B cells using semiquantitative RT-PCR. The cells were treated with L-CoV-2 and ISX shRNA. (**j**) A ChIP assay was conducted using chromatin samples from BEAS-2B and A549 cells with SARS-2-S expression and transfected with or without ISX shRNA. The results are shown as the mean ± s.d., using unpaired t-tests based on three to five biological replicates (**a** and **f**-**j**)

ISX, a pro-inflammatory cytokine (or virus)-inducible gene that transcriptionally regulates downstream tyrosine catabolism enzymes, such as the indoleamine 2, 3-dioxygenases (Wang et al. 2017, 2023a). Consequently, it can enhance levels of kynurenine, immune checkpoint regulators (PD-L1 and B7-2), and epithelial–mesenchymal transition (EMT) regulators (Twist1 and Snail1) and thereby affect the survival time of patients with HCC (Wang et al. 2020b). As ISX was one of the differentially expressed genes in the SARS-CoV-2 infected cells, the impact and potential regulatory effects of ISX were evaluated in these cells. BEAS-2B cells were transfected with envelope small membrane protein E, nucleoprotein N, and SARS-2-S encoded in SARS-CoV-2 genome to determine the activation effect on ISX. BEAS-2B cells transfected with SARS-2-S significantly promoted ISX expression in both mRNA and protein level (Figs. 4a–b). To gain further molecular insights into the function of ISX, target genes regulated by ISX in the BEAS-2B cells with SARS-2-S transfection were evaluated using chromatin immune precipitation sequencing (ChIP-seq) with anti-ISX mAbs (Fig. 4c-d and S1e). A peak calling of 100-bp genomic DNA fragments localized within the 1.5-kb promoter region of each target gene was defined as downstream ISX targets (Figure S1f). A total of 421 genes were identified as ISX downstream candidates, including key transcriptional and metabolic regulators. To further characterize these targets, motif enrichment analysis using MEME-ChIP and pathway enrichment analysis using KEGG were performed (Figure S1e-g), revealing significant enrichment of immune and metabolic regulatory pathways, e.g. *VSIR, MYD88, SNAI1, FOSB,* and *MAFF* (Fig. 4d), and metabolic genes clustered in the AA pathway (Fig. 4e). The genomic binding fragments in the promoter regions of key regulators and metabolic genes clustered in the AA pathway are shown in Fig. 4d-e. Most of the peak calling sequences were within the 1-kbp proximal promoter region. Interestingly, inflammatory and/or fibrogenic precursors significantly activated in pulmonary cells transfected with SARS-2-S showed to be abrogated in pulmonary cells with *ISX*-specific shRNA, including *FOS(B), IL-23, MAFF, MyD88, TNFa, VSIR, SNAI1,* and *LTA4* (Figs. 4f-i). As anticipated, ISX exhibited significantly increased genomic binding activity at the promoter regions of inflammatory and/or fibrogenic precursor genes in pulmonary cells transfected with SARS-2-S. Moreover, these SARS-2-S–induced ISX genomic binding activities were abrogated in pulmonary cells expressing ISX-specific shRNA, affecting targets such as *FOS(B), IL-23, MAFF, MyD88, TNFα, VSIR, SNAI1,* and *LTA4* (Fig. 4j).

### ISX expression is essential to promote immune suppression and AA metabolic pathway induced by SARS-2-S in pulmonary cells

**Fig. 5:**
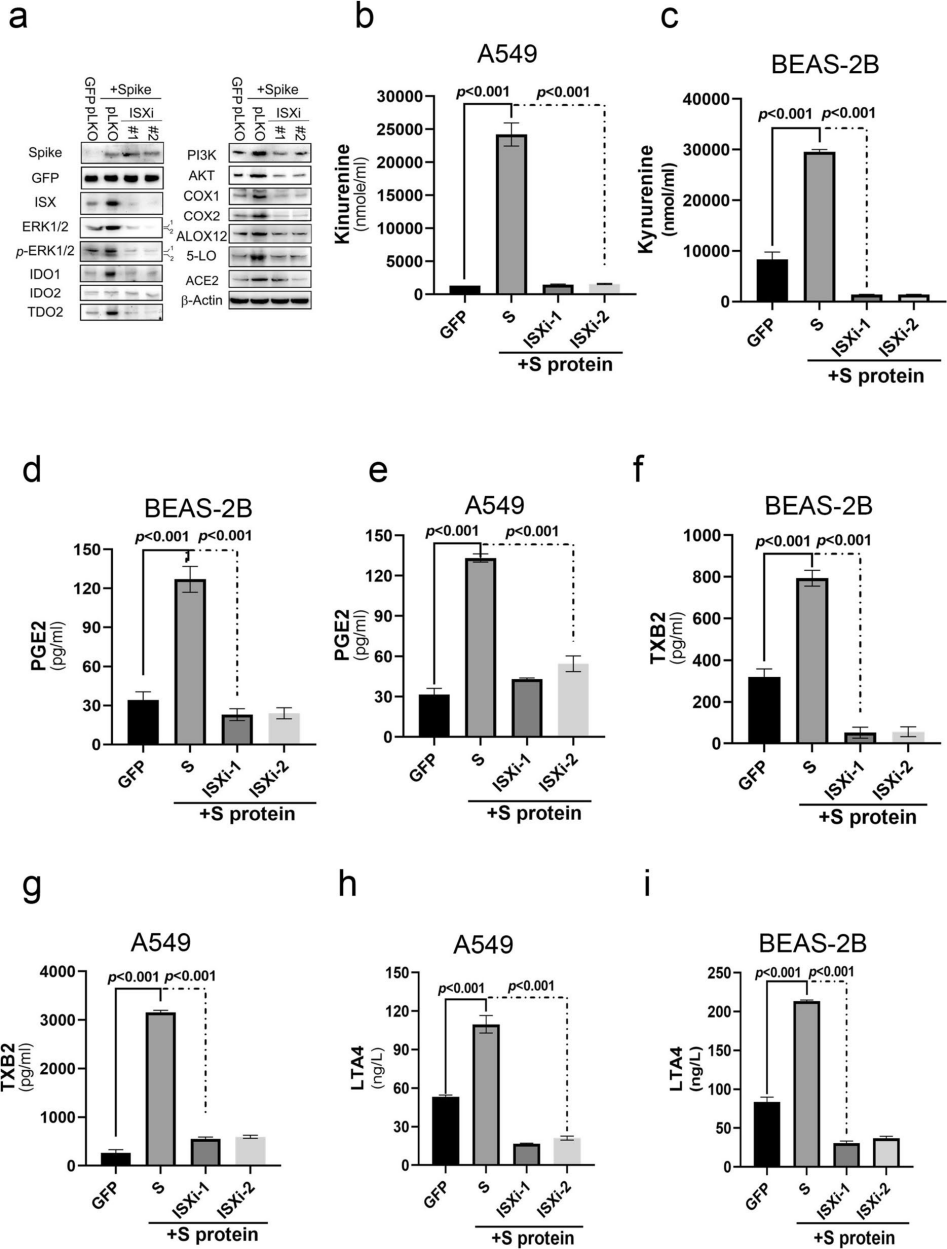
SARS-2-S regulates the AA pathway by ISX (**a**) Protein expression levels of immune co-inhibitors and AA signaling pathway were determined in SARS-2-S expressing BEAS-2B cells transfected with ISX shRNAi or sham control. (**b**–**i**) Kynurenine, PGE2, LTA4, and TXB2 levels were measured in the culture medium of cells. The results are shown as the mean ± s.d., using unpaired t-tests based on three to five biological replicates

To investigate the regulatory mechanisms by which SARS-2-S influences ISX signaling, particularly in relation to immune function and the arachidonic acid (AA) pathway, pulmonary cells were co-infected with L-CoV-2 or co-transfected with GFP-tagged SARS-2-S and ISX-specific shRNA. First, pulmonary cells were either infected with L-CoV-2 alone or co-transfected with *ISX*-specific shRNA. Evidence showed that transfection with ISX-specific shRNA abolished the L-CoV-2–induced elevation of metabolites (Figure S2a). In addition, expression of SARS-2-S in pulmonary cells increased protein levels of ISX, tryptophan catabolic enzymes (IDOs), and key metabolic enzymes in the arachidonic acid (AA) pathway (Fig. 5a and Figure S2b). However, this increase induced by SARS-2-S was abrogated in *ISX*-silenced pulmonary cells compared with those seen in mock-transfected cells (Fig. 5a and Figure S2b). Similarly, while SARS-2-S expression significantly increased kynurenine levels and arachidonic acid (AA) metabolites, including PGE₂, TXB₂, and LTA₄, in pulmonary (BEAS-2B and A549) cells, ISX silencing with ISX-specific shRNA markedly reduced kynurenine accumulation and reversed these metabolic increases. (Figs. 5b–i).

### ACE2-MYD88 signal induced by SARS-2-S protein activates ISX-kynurenine-AA metabolism signals through NF-κb signaling pathways

**Fig. 6:**
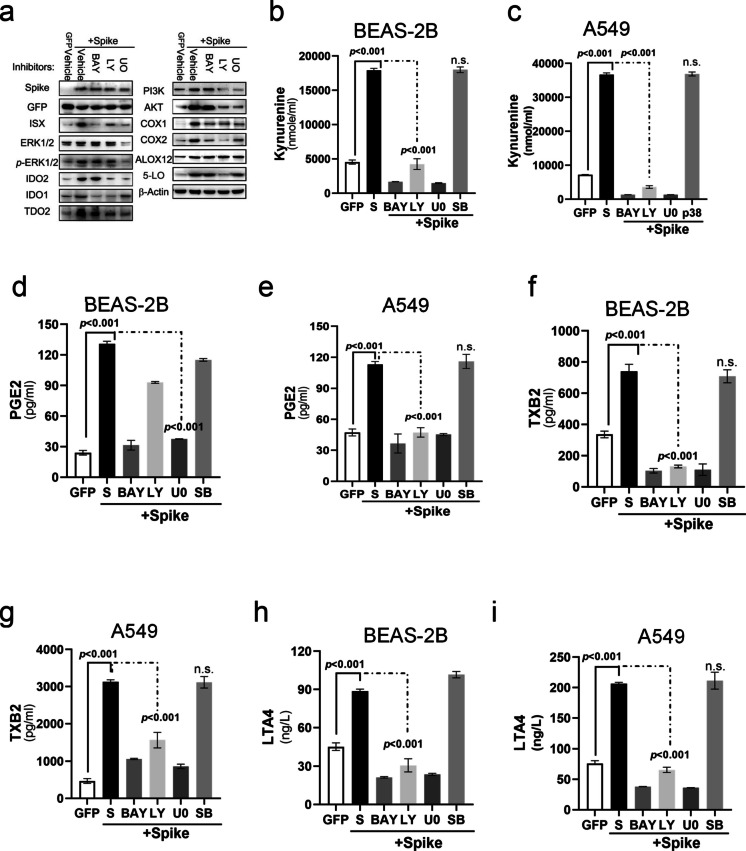
SARS-2-S induces ISX expression, immune suppression, and the AA pathway through the NF-κB, p38, PI3K, and MAPK signaling pathways. (**a**) Western blotting of ISX, immune co-inhibitors, and AA relative signaling protein expression in BEAS-2B cells treated with SARS-2-S and kinase inhibitors. (**b**–**i**) Culture medium of Kynurenine, PGE2, LTA4, and TXB2 detected in BEAS-2B and A549 cells treated with SARS-2-S and kinase inhibitors. The results are shown as the mean ± s.d., using unpaired t-tests based on three to five biological replicates

Pulmonary cells infected with L-CoV-2 or transfected with SARS-2-S were co-incubated with various kinase-specific inhibitors to assess their effects on ISX–kynurenine–AA metabolic signaling. The results showed that both the NF-κB inhibitor (BAY 11–7082, 10 μM) and the MAPK inhibitor (U0126, 5 μM) suppressed L-CoV-2–induced metabolite upregulation (Figure S2c). Next, we analyzed the protein levels of ISX, immune modulators, and enzymes involved in the AA metabolic pathway by western blotting in pulmonary cells transfected with SARS-2-S and co-incubated with various kinase-specific inhibitors. The enhancement of protein expression level in ISX, tryptophan catabolic enzymes, and enzymes involved in the AA metabolic pathway induced by SARS-2-S was significantly suppressed by the NF-κb-specific inhibitor, BAY 11–7082 (10 μM) and the ERK1/2-specific inhibitor, U0126 (an ISX phosphorylation inhibitor (Wang et al. 2021); 5 μM) (Fig. 6a and Figure S2d). Treatment with a PI3K-specific inhibitor, Ly294002 (20 μM), resulted in mild inhibition of the increase in protein levels of ISX, tryptophan catabolic enzymes, and enzymes involved in the AA metabolic pathway; however, treatment with a p38 kinase-specific inhibitor, SB20358 (20 μM) did not affect the levels of these proteins. Treatment of pulmonary cells expressing SARS-CoV-2 Spike with the NF-κB inhibitor BAY 11–7082 (10 μM) or the ERK1/2 inhibitor U0126 (an ISX phosphorylation inhibitor (Wang et al. 2021), 5 μM) markedly reversed the SARS-2-S–induced increase in KYN, PGE2, TXB2, and LTA4 levels. Consistently, inhibition of NF-κB or ERK1/2 suppressed ISX and major metabolic enzymes, whereas certain targets such as IDO1/2 and ALOX12 were less affected (Figs. 6b–i).

### VSIR-ERK1/2 signals induced by SARS-2-S protein activates ISX-kynurenine-AA metabolism signals

**Fig. 7:**
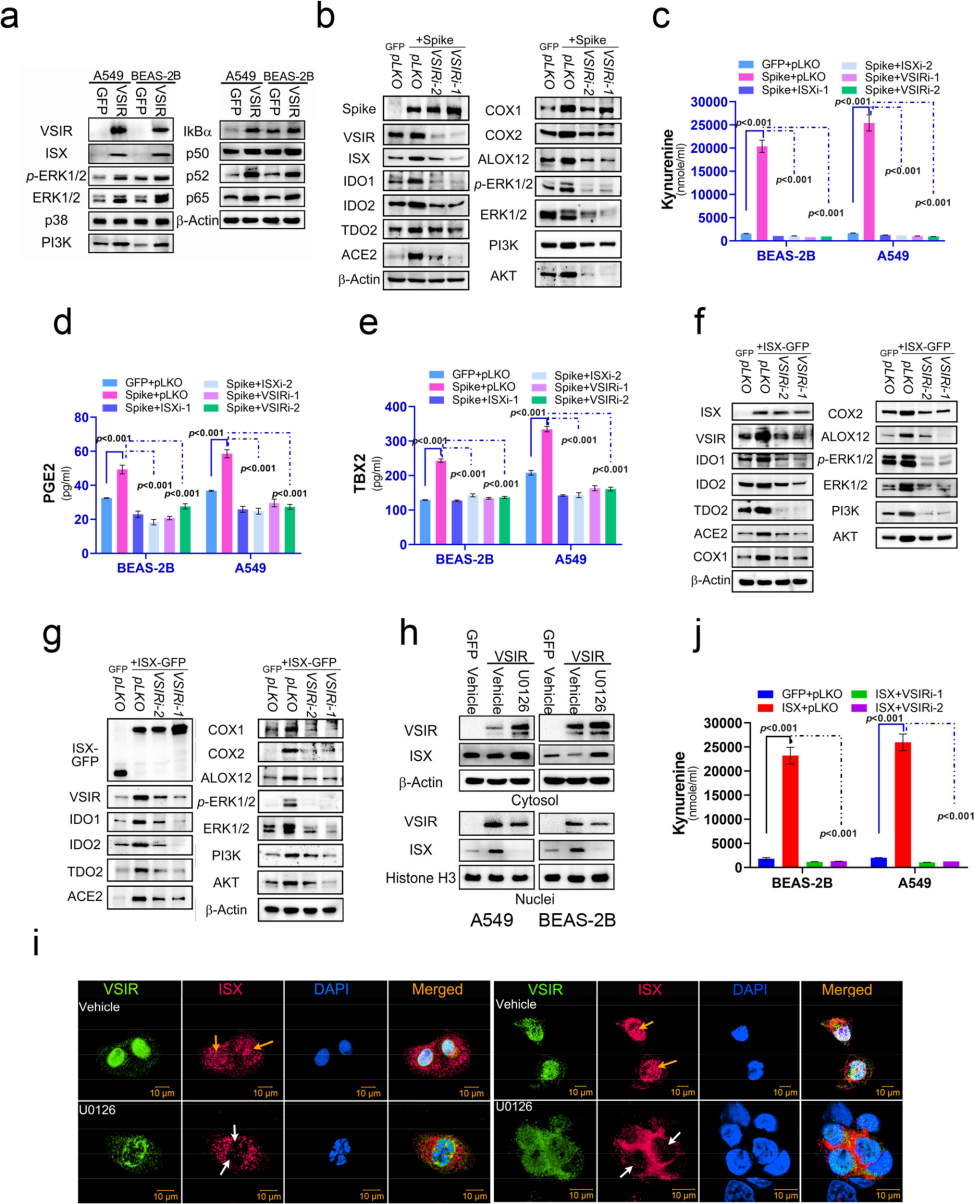
VSIR-ERK1/2 signaling induced by the SARS-2-S protein activates ISX-kynurenine-AA metabolic pathways. (**a**) The protein levels of VSIR, ISX, ERK1/2, p-ERK1/2, p38, PI3K, IκBα, p50, p52, and p65 in BEAS-2B cells were analysed using Western blot. The analysis included BEAS-2B and A549 cells with and without VSIR expression. (**b**) The protein levels of Spike, VSIR, ISX, ERK1/2, p-ERK1/2, IDO, IDO2, TDO2, PI3K, AKT, COX1, COX2, and ALOX12 in BEAS-2B cells were analyzed using Western blot in SARS-2-S-expressing BEAS-2B cells transfected with either VSIR shRNA or control. (c-e) Kynurenine, PGE2, and TXB2 were detected in the culture medium of BEAS-2B and A549 cells treated with SARS-2-S and either ISX or VSIR shRNA. (**f**-**g**) The protein levels of Spike, VSIR, ISX, ERK1/2, p-ERK1/2, IDO, IDO2, TDO2, PI3K, AKT, COX1, COX2, and ALOX12 in BEAS-2B (**f**) and A549 (**g**) cells were analyzed using Western blot in ISX-expressing BEAS-2B and A549 cells transfected with either VSIR shRNA or a control. (**h**) Endogenous ISX levels in cytoplasmic and nuclear fractions were detected in cells transfected with GFP-tagged VSIR, either alone or in combination with U0126. (**i**) Confocal immunofluorescent imaging of ISX and VSIR was conducted in BEAS-2B (left) and A549 (right) cells. ISX was visualized in magenta, VSIR in green, and DAPI was used to indicate nuclei. Yellow and blank arrows indicate nuclear and cytosolic localization of ISX, respectively. (**j**) Kynurenine was detected in the culture medium of ISX-expressing BEAS-2B and A549 cells transfected with either VSIR shRNA or a control. The results are shown as the mean ± s.d., using unpaired t-tests based on three to five biological replicates (**c**-**e** and **j**)

To investigate the regulatory effects of VSIR induced by the SARS-CoV-2 spike (S) protein, pulmonary cells transfected with VSIR-GFP were analyzed for key markers in relevant signaling pathways (Fig. 7a and Figure S2e). The results demonstrated that VSIR-GFP transfected pulmonary cells exhibited a significant upregulation in MAPK signaling (phospho- and total ERK1/2) and NF-κB signaling (p65 and p52) (Fig. 7a and Figure S2e). Notably, VSIR expression markedly activated ISX expression (Fig. 7a and Figure S2e). Conversely, pulmonary cells transfected with *VSIR*-specific shRNA showed suppression of the upregulation in ISX-kynurenine-AA metabolism signaling induced by the SARS-CoV-2 S protein (Fig. 7b and Figure S2f). This suppression extended to the levels of kynurenine-AA metabolism metabolites (kynurenine, PGE, and TXB2) in these cells (Figs. 7c-e). Additionally, *VSIR*-specific shRNA transfection inhibited the upregulation of MAPK signaling and kynurenine-AA metabolism induced by ISX (Figs. 7f-g and Figure S3a-b).

Interestingly, treatment with an ERK1/2 inhibitor (U0126, 5 μM) in pulmonary cells suppressed the nuclear localization of ISX induced by VSIR expression (Fig. 7h and S3c). Confocal microscopy revealed that ISX (green) exhibited pronounced nuclear localization in VSIR-transfected pulmonary cells (yellow arrow). In contrast, in cells treated with the ERK1/2 inhibitor (U0126), ISX displayed a cytosolic expression pattern (blank arrow) (Fig. 7i). Finally, *VSIR*-specific shRNA transfection also suppressed the upregulation of kynurenine-AA metabolism metabolites (kynurenine, PGE, and TXB2) induced by ISX (Fig. 7j).

## Discussion

COVID-19 is an ongoing pandemic infection caused by the novel coronavirus (SARS-CoV-2) and has taken a major toll on human life(Li et al. 2020). While advances in vaccination have significantly alleviated acute symptoms, the long-term effects of SARS-CoV-2 infection and the associated therapeutic strategies remain inadequately understood. This study demonstrates that the SARS-CoV-2 Spike (S) protein perturbs tryptophan and arachidonic acid metabolic pathways through activation of the VSIR–ISX signaling axis, leading to increased production of immunomodulatory metabolites such as kynurenine, TXB2, PGE2, and LTA4. Mechanistically, the ACE2-MYD88 signaling pathway, activated by the SARS-CoV-2 S protein, enhances the VSIR-ISX axis through NF-κB signaling, leading to metabolic disturbances, particularly in tryptophan and AA metabolism, and exacerbating disease progression. These metabolic disruptions contribute to inflammation and immune suppression. The findings of this study offer potential insights for developing new therapeutic approaches against COVID-19 in the future.

AA signals are polyunsaturated fatty acids produced by membrane phospholipids through phospholipase-A2 (PLA2) in inflammatory condition (Ripon et al. 2021). In cells, AA also acts as an endogenous antiviral compound inducing the inactivation of enveloped viruses, including influenza virus and SARS-CoV-2 (Ma and Turk 2001). AA metabolic pathway induced the uncoupling of oxidative phosphorylation in mitochondrial respiratory chain reaction and promoted the lysis of microbial cell membranes (Brash 2001; Pompeia et al. 2000). The bioactive mediators in AA metabolism regulate cardiovascular disease, carcinogenesis, and many inflammatory diseases, such as asthma, arthritis, cancers, etc. (Beck et al. 1998; Bazan et al. 1990). Many mediators, such as prostanoids, LTs, ETEs, and LXs, are considered novel preventive and therapeutic targets for the above diseases (Korotkova and Lundberg 2014). In this study, evidence showed SARS-CoV-2 infection led to a significant increase in the serum levels of PGE2 and TXB2, especially, while decreasing the level of LTA4 in patients with both mild and severe symptoms when compared to individuals without SARS-CoV-2 infection. TXB2, a stable metabolite of thromboxane A2 derived from arachidonic acid via the cyclooxygenase pathway, serves as a marker of thromboxane synthesis and platelet activation. It plays critical roles in vasoconstriction, platelet aggregation, and inflammatory responses, although there is currently no evidence supporting a direct association between TXB2 and COVID-19 pathogenesis (Mohajeri and Cicero 2024; Sundaravadivel et al. 2021). Dysregulation of TXB2 can have significant implications for various diseases, including cancer and developmental disorders (Sun and Liu 2023; Sheeba and Logan 2017; Ashaie and Chowdhury 2016). Also, evidences further show the SARS-2-S activates inflammatory (or virus)-induced proto-oncogene-ISX expression (Wang et al. 2023b) to enhance AA metabolism and change levels of downstream bioactive mediators, including PGE2 and TXB2, elevated in patients with SARS-CoV-2 infection. These changes lead to inflammation and immune interference. The evidence in this study further clarifies the importance of the SARS-2-S in SARS-CoV-2 infections.

ACE2 receptors, a specific homolog of the angiotensin-converting enzyme, generally cut up the angiotensinogen into angiotensin II to increase blood pressure and inflammation (Simko and Baka 2022; Wosten-van Asperen et al. 2011). This increases damage to blood vessel linings and tissues. ACE2 binds to the SARS-CoV-2 Spike protein in the respiratory tract and lungs, facilitating viral entry through endocytosis and contributing to COVID-19 pathogenesis (Bai et al. 2016; South et al. 2020). Also, activated ACE2 enhanced angiotensin II levels and Ang II type 1 receptor (AT1R) activity (a G-protein coupled receptor) results in NF-κB signaling, cytokine storm, and macrophage activation syndrome (Zhu et al. 2015). Blocking ACE2 with specific shRNAi or inhibiting NF-κB signals abolished the AA and tryptophan metabolic disturbance induced by SARS-CoV-2 virus infection in lung cells (Li et al. 2016; Yan et al. 2021). In this study, we demonstrated that activation of NF-κB signaling by the SARS-CoV-2 Spike protein is driven by the interaction between ACE2 and MYD88, clarifying a key signal transduction pathway underlying SARS-CoV-2–induced immune and metabolic dysregulation. Importantly, Delta and Omicron variants harbor multiple mutations within the receptor-binding domain that increase ACE2 binding affinity and potentiate downstream MYD88–NF-κB signaling. This enhanced signaling cascade amplifies VSIR–ISX activation and arachidonic acid (AA) pathway flux, resulting in stronger immunomodulatory and metabolic responses compared to the Beta variant (Figs. 2d–e). These findings underscore the mechanistic importance of ACE2–MYD88–NF-κB signaling in mediating variant-specific differences in host immune activation during COVID-19.

COVID-19 evades immune detection by suppressing human immune responses (Kasuga et al. 2021; Catanzaro et al. 2020). The presence of disequilibrium between pro-inflammatory cytokines and checkpoints in SARS-CoV-2 virus infection may be associated with the expression of proto-oncogene-ISX and relevant IDOs (IDO1 and IDO2)-Kynurenine pathway activation. Intestine- specific homeobox (ISX) is a pro-inflammatory cytokine transcription factor inducible via cellular NF-κB signals (Wang et al. 2023b; Hsu et al. 2013). The ISX expression strike enhanced the levels of tryptophan catabolic enzymes, indoleamine 2,3-dioxygenase 1 (IDO1), tryptophan 2,3-dioxygenase, and immune checkpoints regulators PD-L1 and B2-7 (CD86) in hepatocellular carcinoma cells (Wang et al. 2017). This resulted in partially, at least, an ISX-dependent increase in the tryptophan catabolite kynurenine and immune escape. SARS-CoV-2 infection significantly increased VSIR (an immune checkpoint regulator) and ISX expression through activation of NF-κB signaling. Blocking VSIR or ISX with specific RNAi abolished SARS-2-S–induced inflammation and metabolic disturbances, underscoring the critical role of the VSIR–ISX axis in maintaining immune–metabolic balance during infection. Mechanistically, activation of ACE2–MYD88 signaling by the Spike protein promotes the NF-κB–ISX pathway, leading to arachidonic acid– and tryptophan–related metabolic perturbations that drive inflammation and immune suppression. Notably, the dynamic expression pattern of VSIR (VISTA) observed in COVID-19 patients, as reported by Rendeiro et al. (Tan and Li 2022), suggests stage-dependent immunoregulatory roles—upregulated in CD4⁺ and CD8⁺ T cells during mild/moderate disease to prevent hyperactivation, but reduced in severe cases while remaining above baseline, reflecting a compensatory mechanism following T-cell exhaustion. Together, these findings provide mechanistic insights into how the VSIR–ISX signaling axis links antiviral defense, immune suppression, and metabolic dysregulation, which may inform future strategies aimed at restoring immune–metabolic balance during SARS-CoV-2 infection.

## Materials and methods

### Plasmids and lentivirus construction

Full-length SARS-CoV-2 Spike (S) cDNA was amplified by PCR from the full-length Spike (RBD) clone (Sino Biological, Beijing, China) and subcloned into the pEGFP-C1 vector (Clontech, California, USA) to generate a GFP-tagged Spike expression construct. The SARS-CoV-2 Spike (S) pseudotyped lentivirus (L-CoV-2) was subsequently produced by the RNAi Core Facility (Academia Sinica, Taipei, Taiwan) using a third-generation lentiviral packaging system. Briefly, the codon-optimized full-length Spike sequence (GenBank accession no. NC_045512.2) was inserted into the pLKO.1 vector under the control of a CMV promoter. HEK293T cells were transfected with the Spike-expressing construct, which was generated by subcloning the Spike sequence into the pEGFP-C1 vector (Clontech, USA), using Lipofectamine 3000 (Invitrogen). After 48–72 h, viral supernatants were collected, clarified by centrifugation, and filtered through 0.45-μm membranes. Viral titers were determined by p24 ELISA (Takara) and normalized across experiments.

For infection assays, BEAS-2B and A549 cells were seeded at 70–80% confluence and incubated with L-CoV-2 at a multiplicity of infection (MOI) of 5 in the presence of 8 μg/mL polybrene for 24 h. After infection, cells were washed and cultured for an additional 48 h before RNA and protein extraction. Infection efficiency was validated by GFP fluorescence (for control virus), qPCR detection of Spike transcripts, and immunoblotting for Spike protein expression. Control pseudoviruses carrying GFP or empty pLKO.1 vectors were produced in parallel under identical conditions. The pLKO.1-puro and pLKO.1-neo backbones were also used for constructing shRNA vectors targeting ISX, VSIR, MYD88, and ACE2 (Table S1).

### Cell culture

The human lung cancer cell line, BEAS-2B (RRID:CVCL_0168) and A549 (RRID:CVCL_0023), were purchased from the American Type Culture Collection (ATCC; Manassas, VA, USA) in June 2020. Cell lines from ATCC have been thoroughly tested and authenticated; morphology, karyotyping, and PCR-based approaches were used to confirm the identity of the original cell lines. Cells were grown in LHC-9 or 90% Eagle minimum essential medium (Gibco, Grand Island, NY, USA) with 2 mM L-glutamine and Earle’s balanced salt solution (Gibco) adjusted to contain 1.5 g/L sodium bicarbonate, 0.1 mM nonessential amino acids (Gibco), 1.0 mM sodium pyruvate, and 10% fetal bovine serum (Gibco). All cell lines were routinely tested for mycoplasma contamination using a Universal Mycoplasma Detection Kit (Thermo Fisher Scientific, Waltham, MA, USA), and the last mycoplasma test was performed in March 2024. Mycoplasma-free cell lines were used in all experiments.

### Next-generation sequencing (RNA-Seq) and data analysis

Next-generation sequencing was performed as previously described (Wang et al. 2023b), using the Illumina TruSeq RNA Library Preparation Kit v2 with polyA selection to obtain 50 cycles of single-end reads. The reads were subsequently aligned to the March 2022 human reference sequence genome (GRCh38) using the software Hisat2 (v2.0.1), after which the sample reads were visualized using the Integrated Genome Browser (Version 9.0.1). Next, differentially expressed genes were identified using the DESeq2 Bioconductor package. The DRDS function was used to calculate the false discovery rate (FDR) statistic for the significance of differentially expressed genes. The analysis used a log-transformed FPKM of > 0.1 in at least one treatment group. DEGs with fold changes > 1.5 and only log-transformed FDR < 0.05 were used. Means-centered log-transformed FPKM was used to prepare hierarchical clustering heatmaps in Cluster (version 3.0) and Java Tree View (version 3.0). A final significant differential gene list was used for gene enrichment analysis, including Gene Ontology (Biological Process) and the KEGG pathway.

### Western blotting and immunohistochemical analysis

Western blotting and immunohistochemical (fluorescence) staining were performed as described previously (Hsu et al. 2013; Chiou et al. 2014). The primary antibodies used in this study included Actin (polyclonal, 1:5000 dilution; Sigma–Aldrich, St. Louis, USA) and GFP (monoclonal, 1:500 dilution; Upstate, NY, USA). Additional antibodies were as follows: ISX (GTX49096), IDO1 (Cell Signaling Technology, #86,630), IDO2 (Abcam, ab214214), TDO2 (Abcam, ab204162), PI3K p85 (Cell Signaling Technology, #4257), ERK1/2 (Cell Signaling Technology, #4695), phospho-ERK1/2 (Thr202/Tyr204) (Cell Signaling Technology, #4370), AKT (Cell Signaling Technology, #9272), COX-1 (Abcam, ab109025), COX-2 (Cell Signaling Technology, #12,282), ALOX12 (Abcam, ab211506), and MYD88 (Proteintech, 23230–1-AP) and 5-Lipoxygenase (GTX02838). All experiments were independently repeated at least three times to ensure reproducibility.

### RNA isolation and Real-time PCR

Total RNA from liver tissue RNA was isolated by RNeasy Mini Kit according to the manufacturer’s instructions (Qiagen, Valencia, CA, USA) and then transcribed into cDNA (Invitrogen) for PCR amplification on a STEPONE Thermocycler (Applied Biosystems Inc., Waltham, USA). Semi-quantitative real-time PCR was performed with SYBR Green FastMix (Applied Biosystem Waltham, USA) and analyzed using ΔΔCt calculations. All data were normalized to GAPDH expression. All data are expressed as the mean ± standard deviation (SD) of at least three experiments.

### ELISA analysis

The levels of human kynurenine, PGE2, LTA4, and TXB2 were quantified using commercial ELISA kits according to the manufacturers’ instructions (R&D Systems, Minneapolis, USA; Cusabio, Houston, USA). Culture supernatants were collected after centrifugation and analyzed in triplicate. Concentrations were calculated from standard curves generated with purified standards provided in each kit. The detection limits for the ELISA assays were as follows: kynurenine, 0.5 nmol/mL; PGE2, 15 pg/mL; LTA4, 10 pg/mL; and TXB2 12 pg/mL.

### Bioinformatics analyses for the expression profiles

Gene ontology (GO) enrichment analysis and DAVID annotation were used for functional annotation and pathway analysis, such as molecular function, biological process, and cellular component. The KEGG (https:\\www. genome.jp\kegg\ analyses) was consulted to evaluate the biological function of DEGs. GO terms with FDR (*p* < 0.05) were considered significantly enriched within the gene set.

### Chromatin immunoprecipitation (ChIP)-sequencing and data analysis

ChIP assays from three independent experiments were performed according to the manufacturer’s protocol (Upstate Biotechnology) (Nakato and Sakata 2021). An ISX-DNA complex was prepared in immune precipitants using anti-ISX antibody or IgG (as a negative control). Then, after recovering the DNA from the complex for further analysis, ChIP DNA libraries were sequenced as single 150 bp reads (tags) using the Illumina Hiseq 6000 sequencer (Illumina). Raw reads were trimmed for length (n ≥ 32), quality (Phred score ≥ 25), and adaptor sequence using fastp v0.20.0 (Chen et al. 2018). Twenty-one trimmed reads were then aligned to the human genome (GRCh38) using BWA v0.5.9. The peaks were analyzed using MACS2 software (Feng et al. 2012) (mfold = 10, 30; bandwidth = 300; p-value cutoff = 1E-5) and sequenced using libraries of matched IgG DNA as control (Yu et al. 2015). The fragment length used was the one predicted using the program. Peak list intersections were detected using BEDTools v2.12.0 (Quinlan and Hall 2010).

### Patients

In this retrospective analysis, we included 115 patients (56 men and 59 women; mean age, 61.3 ± 5.89 years; range, 25–82 years) with confirmed COV-19 infection between March 2020 to May 2023 from two medical centers (National Biobank Consortium of Taiwan (100) and Tainan Hospital, Ministry of Health and Welfare (15)). The study was conducted with approval from the Ethics Committee of Kaohsiung Medical University Chung-Ho Memorial Hospital (KMUHIRB-E(I)−20,210,126). Written informed consent was obtained from all participants prior to sample collection. All study procedures were performed in accordance with the ethical standards of the institutional research committee and with the principles of the Declaration of Helsinki.Statistical analysis of categorical variables were carried out by one-way ANOVA; *, *p* < 0.05.

### Statistical analysis

The quantitative variables are presented as mean ± SD. Significant differences were determined using a two-sample t-test. Statistical analysis of categorical variables was performed using χ2 analysis, one-way analysis of variance, and Fisher's exact analysis. Differences with a P value < 0.05 were considered statistically significant.

## Supplementary Information

Below is the link to the electronic supplementary material.

Supplementary file1 (TIF 25097 KB)

Supplementary file2 (TIF 24538 KB)

Supplementary file3 (TIF 23983 KB)

Supplementary file4 (DOCX 13 KB)

## Data Availability

Data supporting the findings of this study are available from the corresponding author upon reasonable request.

## Abbreviations

ISX: Intestine Specific Homeobox
AA: Arachidonic acid
SARS-2-S: SARS-CoV-2 Spike protein
ACE2: Angiotensin-converting enzyme 2
VSIR: V-set immunoregulatory receptor
IC: Immune checkpoint
LTs: Leukotrienes
ETEs: Eicosatetraenoic acids
LXs: Lipoxins
L-Cov-2: SARS-CoV-2 pseudotyped lentivirus
KEGG: Kyoto Encyclopedia of Genes and Genomes
PGE2: Prostaglandin E2
LTA4: Leukotriene X4
TXB2: Thromboxane B2
PLA2: Phospholipase-A2

## Data and code availability

https://www.ncbi.nlm.nih.gov/sra/PRJNA1341786

The data that support the findings of this study are available from the corresponding author [jackhsu@kmu.edu.tw] upon reasonable request.
