# Supplementary figures and images for: Spike protein–induced VSIR–ISX signaling disrupts metabolic homeostasis and promotes COVID-19–related immune dysfunction
Source: Cell Biol Toxicol. 2025 Dec 19;42(1):2. doi: 10.1007/s10565-025-10119-2 (PMC12717150; doi:10.1007/s10565-025-10119-2)

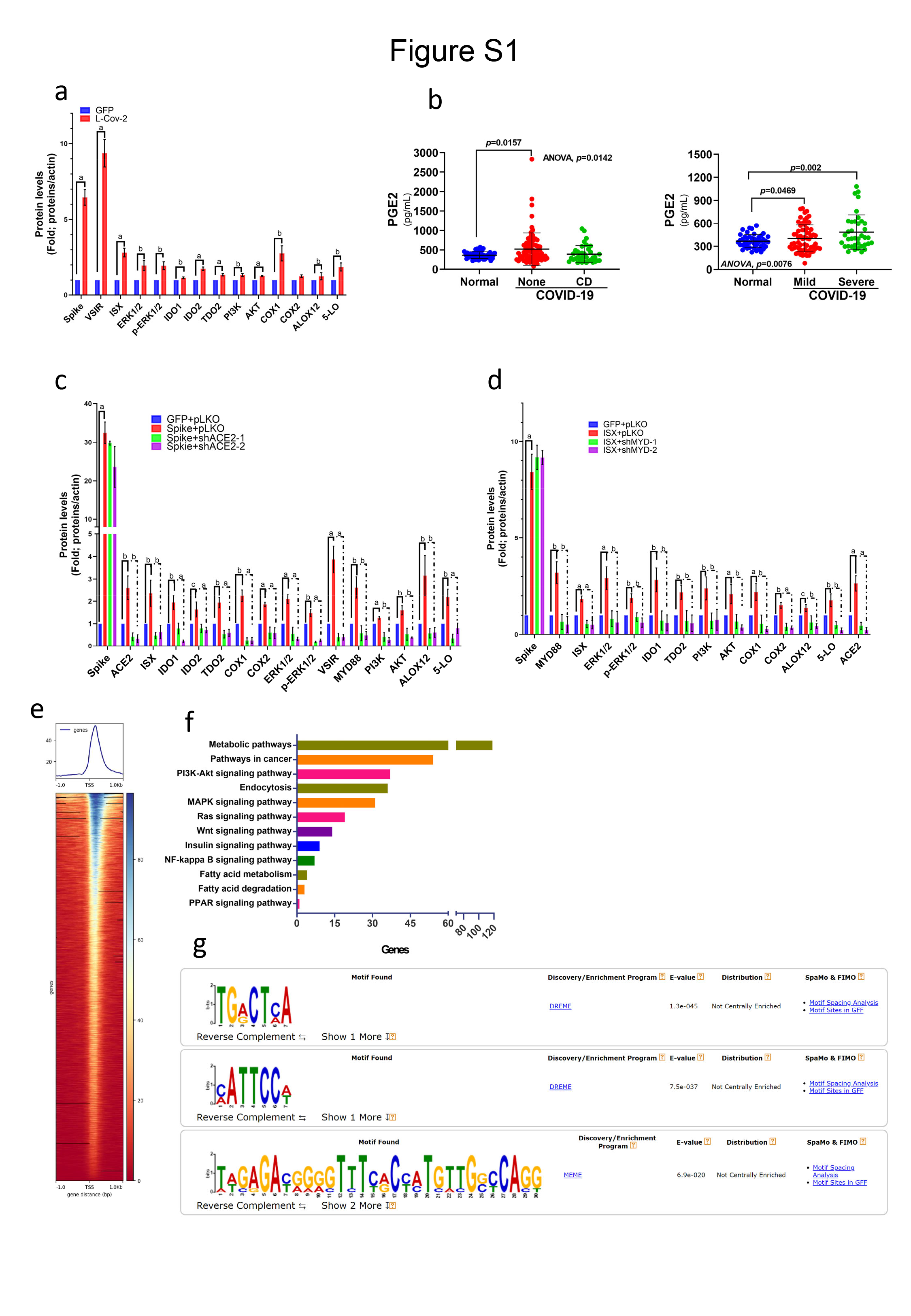

Supplement: Supplementary file 1 — Supplementary file1 (TIF 25097 KB) [file 10565_2025_10119_MOESM1_ESM.tif]

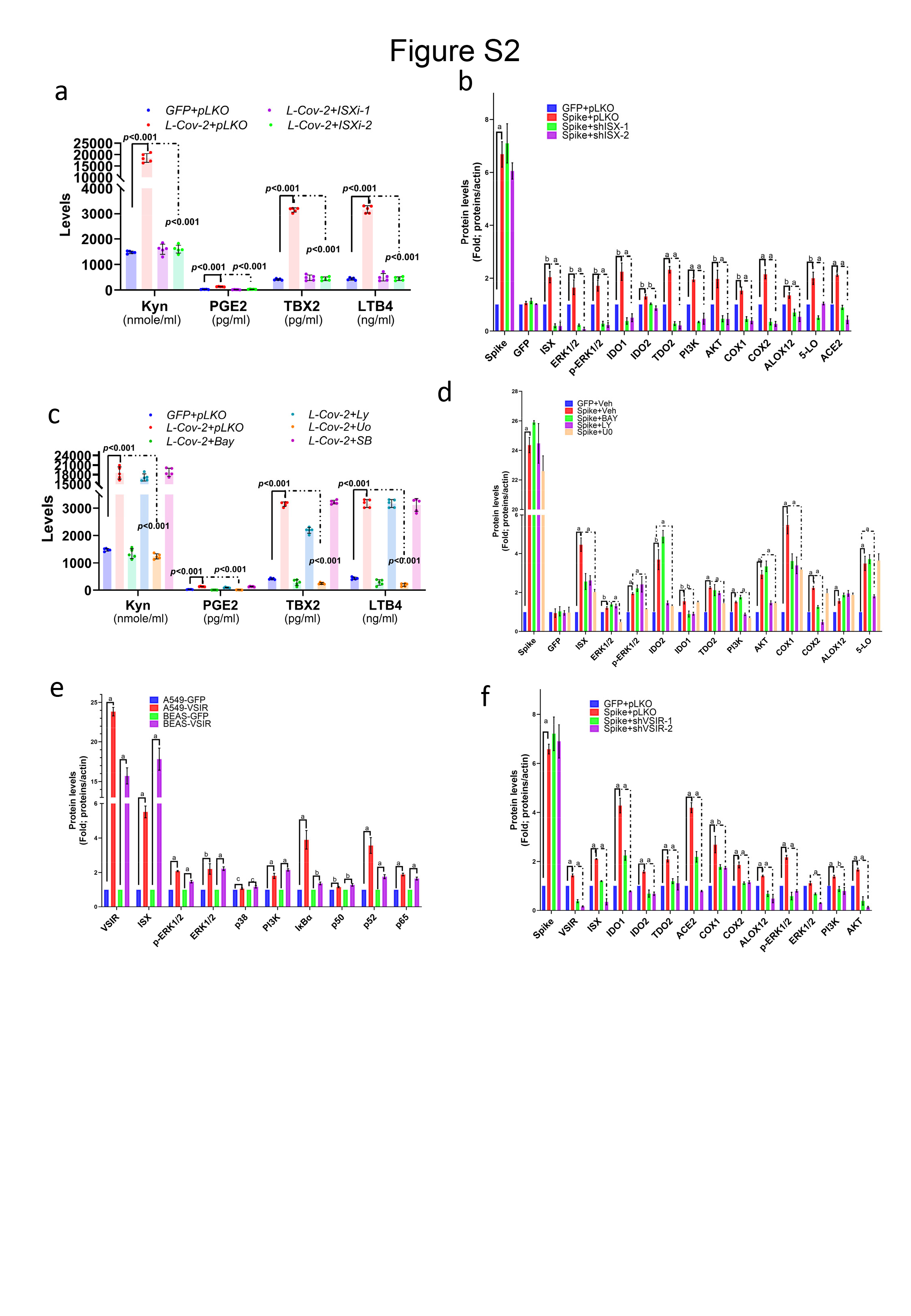

Supplement: Supplementary file 2 — Supplementary file2 (TIF 24538 KB) [file 10565_2025_10119_MOESM2_ESM.tif]

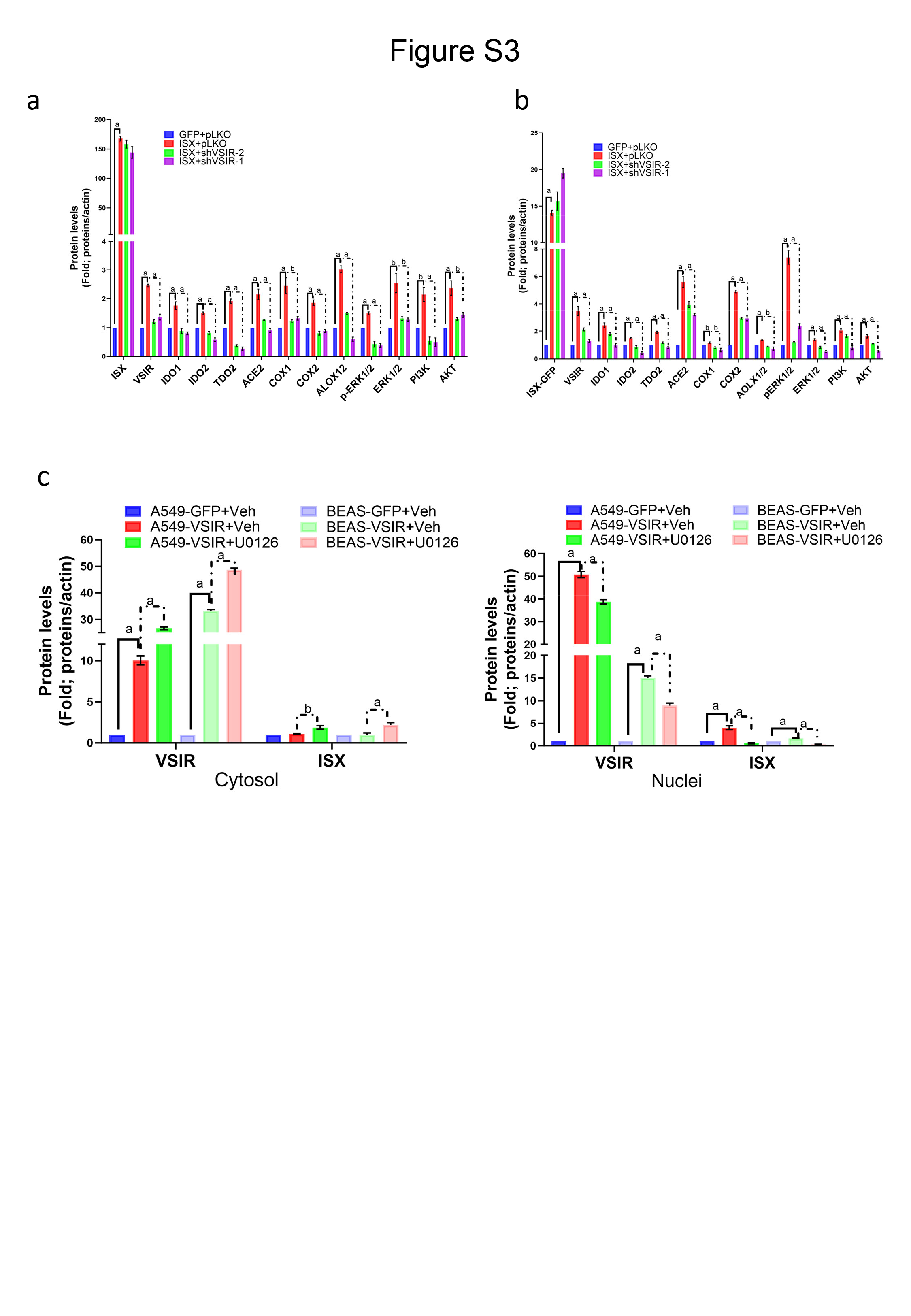

Supplement: Supplementary file 3 — Supplementary file3 (TIF 23983 KB) [file 10565_2025_10119_MOESM3_ESM.tif]
