# Supplementary material for: Spike protein–induced VSIR–ISX signaling disrupts metabolic homeostasis and promotes COVID-19–related immune dysfunction
Source: Cell Biol Toxicol. 2025 Dec 19;42(1):2. doi: 10.1007/s10565-025-10119-2 (PMC12717150; doi:10.1007/s10565-025-10119-2)
Supplement: Supplementary file 4 — Supplementary file4 (DOCX 13 KB) [file 10565_2025_10119_MOESM4_ESM.docx]

Table S1. Target sequence of shRNA

| Gene | Sequence |
| --- | --- |
| ISX shRNA1 | GCAGCATCTGTGCTACTTCAA |
| ISX shRNA2 | CAAACTTGCATCCCTGTGCTA |
| VSIR shRNA1 | CCCTGACTCTCCAAACTTTGA |
| VSIR shRNA2 | CACCAGCTACAGATGCCAAAT |
| MYD88 shRNA1 | GCAGAGCAAGGAATGTGACTT |
| MYD88 shRNA2 | CCTGTCTCTGTTCTTGAACGT |
| ACE2 shRNA1 | GCCCTTATTTACCTGGCTGAA |
| ACE2 shRNA2 | GGGCGACTTCAGGATCCTTAT |
